# A burst of genomic innovation at the origin of placental mammals mediated embryo implantation

**DOI:** 10.1038/s42003-023-04809-y

**Published:** 2023-04-26

**Authors:** Alysha S. Taylor, Haidee Tinning, Vladimir Ovchinnikov, Jessica Edge, William Smith, Anna L. Pullinger, Ruth A. Sutton, Bede Constantinides, Dapeng Wang, Karen Forbes, Niamh Forde, Mary J. O’Connell

**Affiliations:** 1 Discovery and Translational Sciences Department, Leeds Institute of Cardiovascular and Metabolic Medicine, Faculty of Medicine and Health, University of Leeds, Leeds, LS2 9JT UK; 2 School of Biology, Faculty of Biological Sciences, University of Leeds, Leeds, LS2 9JT UK; 3 School of Life Sciences, Faculty of Medicine and Health Sciences, University of Nottingham, Nottingham, NG7 2RD UK; 4 Leeds Fertility, Leeds Teaching Hospitals NHS Trust, York Road, Seacroft, Leeds, LS14 6UH UK; 5 Modernising Medical Microbiology Consortium, Nuffield Department of Clinical Medicine, John Radcliffe Hospital, University of Oxford, Oxford, OX3 9DU UK; 6 LeedsOmics, University of Leeds, Leeds, LS2 9JT UK; 7 Wellcome Centre for Human Genetics, University of Oxford, Oxford, OX3 7BN UK

**Keywords:** Molecular evolution, Molecular medicine

## Abstract

The origin of embryo implantation in mammals ~148 million years ago was a dramatic shift in reproductive strategy, yet the molecular changes that established mammal implantation are largely unknown. Although progesterone receptor signalling predates the origin of mammals and is highly conserved in, and critical for, successful mammal pregnancy, it alone cannot explain the origin and subsequent diversity of implantation strategies throughout the placental mammal radiation. MiRNAs are known to be flexible and dynamic regulators with a well-established role in the pathophysiology of mammal placenta. We propose that a dynamic core microRNA (miRNA) network originated early in placental mammal evolution, responds to conserved mammal pregnancy cues (e.g. progesterone), and facilitates species-specific responses. Here we identify 13 miRNA gene families that arose at the origin of placental mammals and were subsequently retained in all descendent lineages. The expression of these miRNAs in response to early pregnancy molecules is regulated in a species-specific manner in endometrial epithelia of species with extreme implantation strategies (i.e. bovine and human). Furthermore, this set of miRNAs preferentially target proteins under positive selective pressure on the ancestral eutherian lineage. Discovery of this core embryo implantation toolkit and specifically adapted proteins helps explain the origin and evolution of implantation in mammals.

## Abstract (web summary)

Here 13 conserved miRNAs are identified that arose at the origin of placental mammals. Their expression is triggered by early pregnancy cues in a species-specific way, providing insights into the origin and evolution of implantation in mammals

## Introduction

**Fig. 1:**
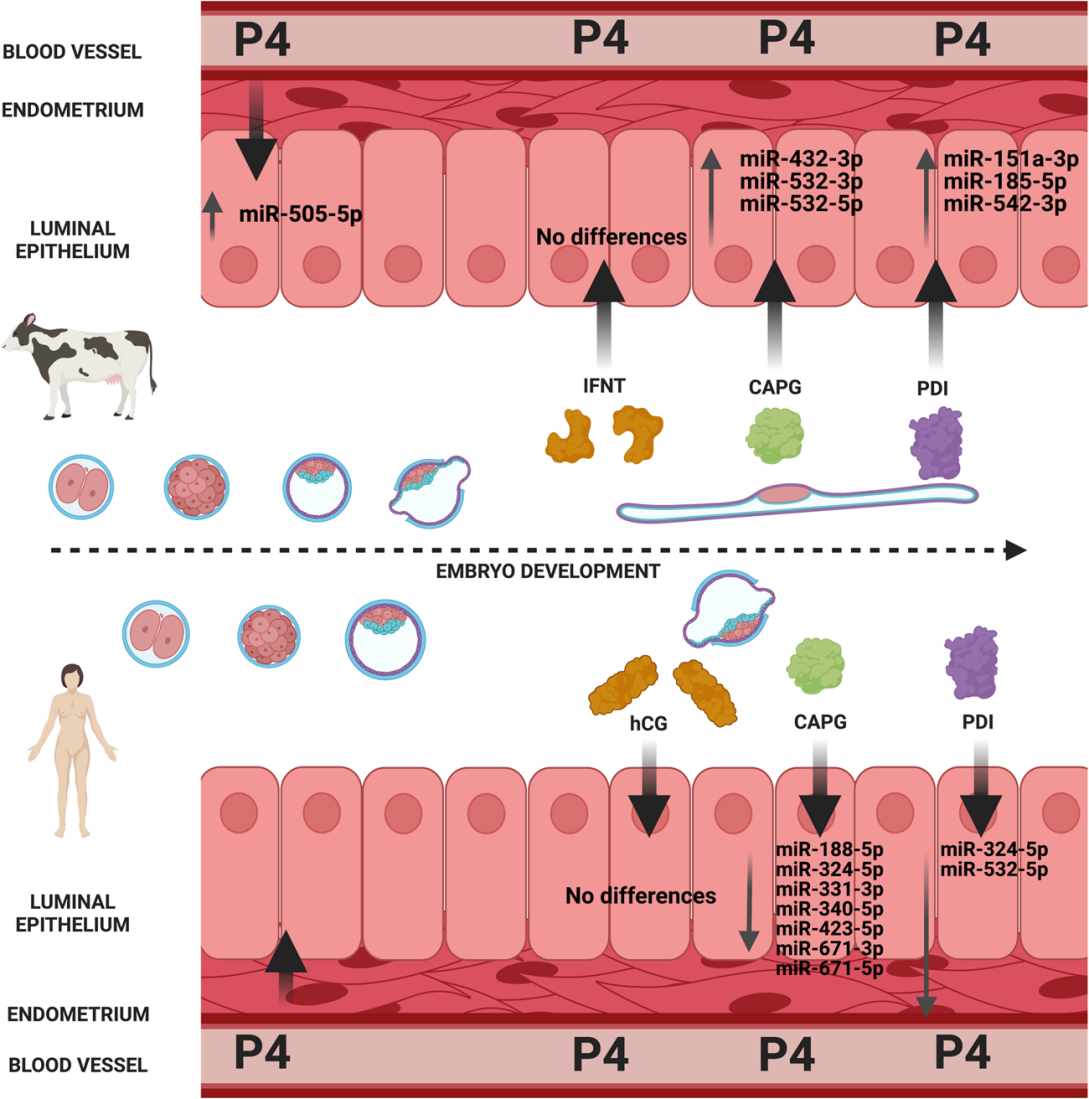
Regulation of core miRNA toolkit by early pregnancy markers. The endometrial epithelium of bovine (superficial implantation strategy) (top panel) or human (invasive implantation strategy) (bottom panel) are regulated by molecules important for endometrial function in early pregnancy in eutheria (P4: progesterone; hCG: human chorionic gonadotrophin; Interferon Tau: IFNT; Macrophage capping protein: CAPG, and protein disulfide isomerase: PDI).

Successful pregnancy in eutheria is contingent on a developmentally competent embryo, appropriate endometrial function, the formation of the placenta, and the molecular cross-talk across these components^1,2^. Yet implantation strategy, placental organ morphology, and more generally, the underlying regulation of successful pregnancy, varies across mammals. Protein coding alterations along with innovations in regulatory networks drive the origin and evolution of novel traits^3^. Moreover, bursts of the evolution of new microRNAs (miRNAs) are known to be associated with morphological innovation^4–6^, and miRNAs are known to regulate placental function in both normal and pathophysiological conditions^7,8^. We have uncovered a core placental toolkit in mammals that evolved through a synergy of molecular events involving the emergence of new miRNAs combined with adaptive amino acid changes in key proteins. We propose that this conserved core toolkit contributes to the diversity of implantation strategies and pregnancy morphologies observed in modern eutheria (Fig. 1).

## Results and discussion

### A core miRNA toolkit of 13 miRNA families arose at the origin of therian and eutherian mammals, were never subsequently lost, have roles in pregnancy and are uterine expressed

**Fig. 2:**
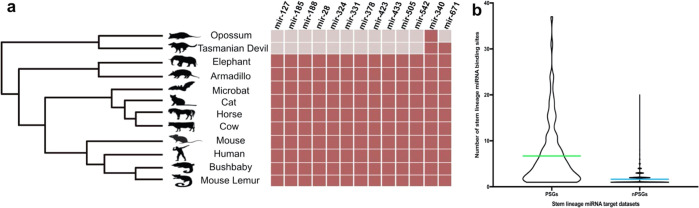
Phylogenetic distribution of the miRNA families and their levels of targeting in positively selected genes. **a** Phylogenetic distribution of miRNA families specific to therian and eutherian mammals. The mammal phylogeny displays the species sampled in our analysis. The corresponding matrix shows the presence (dark red) or absence (pale grey) of miRNA families across the species sampled. **b** Violin plot comparing the number of target miRNA binding sites from the core miRNA toolkit per transcript in the 84 genes that underwent positive selection on the stem Eutherian lineage (PSGs) (left) compared sampled sets of non-positively selected but randomly sampled genes that are targeted by the core toolkit of miRNAs (nPSG) (right). For each of the 84 PSGs, the mean number of predicted miRNA binding sites was determined for each target transcript in humans (depicted in green). This was compared to the mean number of binding sites for each of the nPSGs (depicted in blue). The mean number of binding sites was determined to be significantly different between the two datasets when *p* ≤ 0.05, two-sample *t*-test with unequal variance.

Because significant miRNA family expansions have been found to correlate with major transitions in animal evolution^4–6^, we interrogated MirGeneDB^9^ and identified 112 miRNA families that originated on either the therian (6 miRNA families) or eutherian stem lineage (106 miRNA families). Six of these miRNA families emerged on the older therian stem lineage (mir-340, mir-483, mir-671, mir-675, mir-1251, and mir-3613), of which only mir-340 is phylogenetically conserved in both Theria and Eutheria (Fig. 2a). Given that mir-671 is present in the Tasmanian devil and in all Eutheria sampled, we classified mir-671 as a ‘therian stem lineage miRNA’. A total of 106 miRNAs emerged at the origin of eutheria, and 11 of these remained conserved in all extant eutheria sampled (Fig. 2a). In total, this yielded 13 miRNA families (consisting of 17 miRNA genes) that originated on stem mammal lineages and were subsequently retained in all descendent lineages, henceforth referred to as “the core miRNA toolkit”. All of the miRNAs in the core toolkit are supported by small RNA sequencing data across different mammalian species^9,10^. Expression and function of some of the core miRNA toolkit have been demonstrated for the normal placenta (e.g. mir-433^11^, mir-28^12^, mir-378^13^), and endometrium (e.g. mir-505^14^ and mir-542^15^). Others have been implicated in pathophysiological pregnancies, e.g. mir-185, mir-188, mir-423 and mir-542^16–18^ have been implicated in preeclampsia, mir-127^10^ has been associated with placentomegaly, mir-324 is associated with “large for gestational age” (LGA) pregnancies^19^, mir-331 is associated with placenta from intra-amniotic infection^20^, and mir-505 can be associated with preterm birth^21^. Collectively all 13 of the core miRNA family toolkit have been implicated in critical roles in placental mammal pregnancies. Outside of their role in reproduction, the stem lineage miRNAs have been studied in numerous contexts (Table S1) and have been linked to cancer proliferation and metastasis^22–24^, vascular muscle cell proliferation^25,26^, osteogenesis^27^ and chronic obstructive pulmonary disease^28^.

Targets were identified in the human genome for the core miRNA toolkit using TargetScan^29^. The predicted functions of the targets of the core miRNA toolkit include reproductive functions (55 target transcripts), metabolic process (1476 target transcripts), and biological regulation (1269 target transcripts) (Fig. S1b). More specifically, some of the targets were implicated in pathways associated with INPP5E regulation, Neurophilin interactions, and VEGF interactions with its receptor (VEGR), each involved in the process of angiogenesis. TGF-β signalling and p53 regulation are amongst the predicted targets and are implicated in cell proliferation (Fig. S2). Both angiogenesis and proliferation are required for a successful pregnancy. The syncytins are a family of endogenous retrovirus-derived protein-coding regions that were domesticated in mammals and are essential for promoting placental formation^30^ and 4/13 of the core miRNA toolkit (miR-185-3p, miR-188-3p, miR-423-5p and miR-433-3p) are predicted to target the syncytins with at least 7-mer binding. In the case of miR-423-5p, there are two predicted target sites for *syncytin-1*, one of which has a site overlapping with that of the miR-185-3p-binding site, indicating dynamic/competitive binding between these miRNAs and their *syncytin-1* target. The predicted targets of the core miRNA toolkit also include 130 gene families that have been proposed to have emerged on the Eutherian stem lineage^31^. In addition, the targets included genes that evolved endometrial expression on the stem eutherian lineage, and that are hypothesised to have assisted in the remodelling of the uterine landscape during the evolution of the mammal pregnancy^1^.

Examining the target profile of transcripts that gained uterine expression on the therian and eutherian nodes^1^, we show that 585/1167 transcripts on the therian node are targeted by the therian-derived miRNAs, and 625/835 transcripts on the eutherian node are targeted by the eutherian derived miRNAs. An additional 23 transcripts that gained uterine expression on the eutherian node are targeted by the therian-derived miRNAs (Supplementary Data 2).

### Evidence of positive selection on the stem eutherian lineage

Protein coding alterations (such as the birth of new genes and gene families, gene loss, co-option, and selective pressure variation) along with innovations in regulatory networks drive the development of novel traits^3^. A number of cases of positively selected amino acids (indicative of protein functional shift) are known to have had a direct role in endometrial function, e.g. the galectin family of proteins involved in immune modification at the maternal–foetal interface^32^. We tested for signatures of adaptive evolution in single gene orthologous families (SGOs) on the stem eutherian lineage. We focussed on SGOs to optimise our ability to accurately trace evolutionary histories. We chose a total of ten Eutheria that demonstrate the greatest range of diversity in implantation strategy and placental morphology, plus four outgroup species, one from each of the *Monotremata, Marsupialia, Aves* and *Teleostei* (Table S2). Annotated gene families were taken from Ensembl 90^33^. Following our filtering regime we extracted a total of 1437 SGOs. Applying codon models of evolution to these SGOs, we identified signatures of positive selection on amino acid residues on the stem eutherian lineage in 237 SGOs. The functions of these genes are predominantly cellular processes, metabolic processes, and biological regulation (Fig. S3). Out of these 237 positively selected SGOs, 115 contained positively selected amino acid residues that were subsequently unaltered in all descendent lineages. The 115 SGOs are functionally enriched for chromosomal maintenance, telomere activity, p53 signalling, cell cycle, and the inflammatory immune response—activities that have been associated with the formation of the placental tissue in pregnancy^34–36^. We then studied whether there is a significant association between the core miRNA toolkit and stem-lineage positively selected proteins.

### The core miRNA toolkit preferentially targets genes under positive selection in the stem eutherian lineage

Synergy between regulatory and protein-coding innovations often drives substantial phenotypic novelty^2,3,37^. Therefore, to test the hypothesis that innovation both at the level of regulation and of protein-coding change underpinned the origin of placentation in mammals, we performed a simulation study on the targets of the 17 miRNA genes from the 13 stem lineage miRNA families. Out of 115 SGOs with evidence of positive selection on the ancestral eutherian lineage, 84 were found to be significantly enriched for binding sites for the 13 core miRNA family toolkit (*p* = 1.35618e−11), with a mean of 6.66 binding sites per transcript (median = 4.0) (Fig. 2b). We determined if the number of binding sites in this subset of 84 positively selected SGOs was significantly different than one would expect in comparison to other genes also targeted by these miRNAs but not under positive selection on the ancestral eutherian branch. We estimated the number of binding sites per transcript for the core miRNA toolkit in 100 randomly chosen and non-positively selected gene sets and found it is significantly lower, with a mean = 1.64 (median = 1.0). Assessing the variation in UTR length between these two populations of genes reveals that the non-positively selected set has longer UTRs and that the median UTR length for both was <2 kbp (Fig. 2b). This suggests that the 13 miRNA families in the core miRNA toolkit (a total of 17 miRNA genes) preferentially target the positively selected SGOs (6.66 binding sites compared to 1.64) (Fig. 2b). Combined, this indicates that a co-evolutionary process arose in a short window of time in early mammal evolution that resulted in altered protein function, as well as a new miRNA-mediated regulatory network.

The functions of the 84 positively selected SGOs targeted by the core miRNA toolkit broadly fall within the categories of the cell cycle, DNA damage & DNA metabolic processes, and regulation of hair cycle & hair cycle—a unique mammal characteristic (Fig. S2). We also determined that 21/84 SGOs are significantly more likely to interact with one another (*p* < 0.05) in comparison to any other gene in the genome (Fig. S3b). Of course, there is a significant variation found in modern mammals in other facets such as telomere biology, cancer incidence, body mass and maximum lifespan^38–41^, therefore innovation at this node was not expected to be entirely skewed to implantation and pregnancy (Table S3).

### Species-specific regulation of the core miRNA toolkit by key early pregnancy molecules in species with different implantation strategies

Implantation in eutherian mammals displays wide variation in both embryological morphology and bi-lateral signalling between the embryo and maternal endometrium, and degree of invasiveness (invasive in humans, superficial in bovine). This variation is governed, in part, by conserved signalling pathways, e.g. the sustained actions of the hormone progesterone (P4)^42^, but also by diverse molecular cues such as the maternal recognition of pregnancy signal, e.g. chorionic gonadotrophin (hCG) in human and interferon (IFNT) in bovine^43,44^. We asked the question of what molecules that are involved in bi-lateral communication between the embryo and endometrium, regulate the core mammal miRNA toolkit, and if they are regulated in a species-specific manner. We cultured endometrial epithelial cells from human and bovine^45^ and exposed them for 24 h to P4, recombinant forms of conserved (CAPG, and PDI) or diverse (IFNT and hCG) molecular cues important for early pregnancy success in placental mammals. CAPG and PDI have recently been identified as produced by the bovine conceptus during pregnancy recognition and are highly conserved (in terms of sequence identity and phylogenetic distribution) across placental mammals^45,46^. The sequence identity of the known interacting protein partners of PDI and CAPG is similarly highly conserved across humans and bovines (Table S4). We then examined the expression of the 17 miRNA genes from the 13 stem lineage miRNA families in these cells using a locked nucleic acid (LNA)-based approach. Treatment of bovine endometrial epithelial cells with 10 μg/mL of P4 (recapitulating in vivo P4 exposure during the early luteal phase of the cycle) resulted in increased expression of miR-505-5p (Fig. S4). In summary, treatment with the evolutionarily conserved early pregnancy proteins (P4, CAPG and PDI) in the endometrial epithelial cells of bovine and/or humans resulted in a change in expression of 12/13 of the stem lineage miRNA families (Fig. 1).

**Fig. 3:**
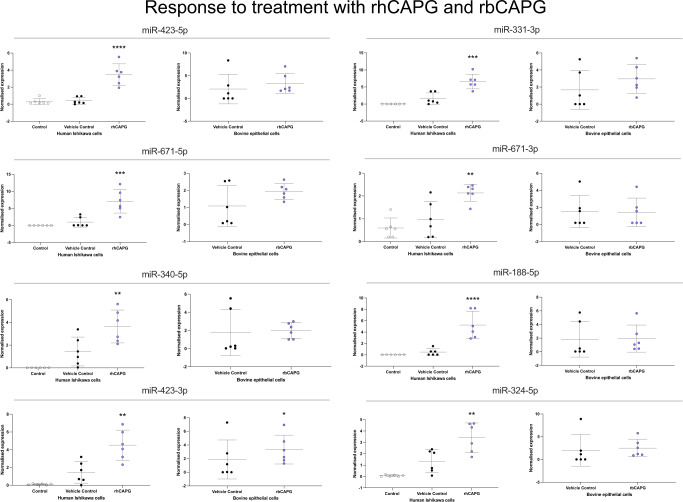
Regulation of stem lineage miRNAs in human and bovine endometrial epithelial cells treated with hCAPG or bCAPG respectively. Expression of stem lineage miRNAs miR-432-5p, miR331-3p, miR-671-5p, miR-671-3p, miR-340-5p, miR-188-5p, miR432-3p and miR-324-5p in either human (left-hand side of each pair) or bovine (right-hand side of each pair) endometrial epithelial cells (*n* = 6 per treatment). Primary bovine endometrial epithelial cells were treated with vehicle control (grey circle), or 1000 ng/μL bCAPG (purple circle) for 24 h. Human Ishikawa immortalised endometrial epithelial cells were treated with control (open circle), vehicle control (closed circle), or 1000 ng/μL hCAPG (purple circle) for 24 h. Significant differences in miRNA expression values determined when *p* ≤ 0.05 are depicted by an asterisk (*).

**Fig. 4:**
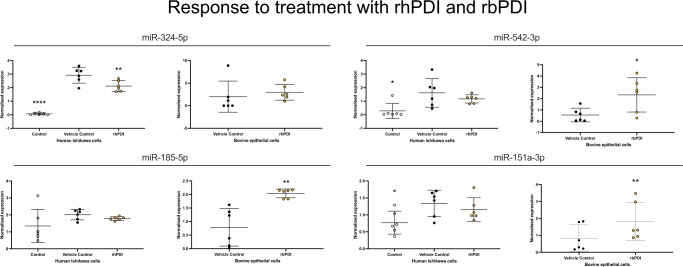
Regulation of stem lineage miRNAs in human and bovine endometrial epithelial cells treated with hPDI or bPDI, respectively. Expression of stem lineage miRNAs mir-324-5p, miR-542-3p, miR-185-5p, and miR151a-3p in either human (left-hand side of each pair) or bovine (right-hand side of each pair) endometrial epithelial cells (*n* = 6 per treatment). Primary bovine endometrial epithelial cells were treated with vehicle control (grey circle), or 1000 ng/μL bPDI (orange circle) for 24 h. Human Ishikawa immortalised endometrial epithelial cells were treated with control (open circle), vehicle control (closed circle), or 1000 ng/μL hPDI (orange circle) for 24 h. Significant differences in miRNA expression values determined when *p* ≤ 0.05 are depicted by an asterisk (*).

Intriguingly, the addition of species-specific recombinant forms of CAPG and PDI proteins to bovine or human endometrial epithelial cells altered the expression of selected miRNAs in a species-specific manner (Figs. 3 and 4, respectively). Treatment of bovine cells (*n* = 6) with recombinant bCAPG resulted in increased expression of miR-432-3p, miR-532-5p, and miR-542-3p in endometrial epithelial cells (*p* < 0.05). Whereas in human endometrial epithelial cells treated with 1000 ng/μL hCAPG (*n* = 6), the expression of miR-188-5p, miR-324-5p, miR-331-3p, miR-340-5p, miR423-5p, miR-671-3p, miR-671-5p increased significantly (*p* < 0.05) compared to controls (Figs. 3 and S7).

Treatment with recombinant bPDI decreased expression of miR-151a-3p, miR-185-5p, and miR-542-3p in bovine epithelial cells (*p* < 0.05: Fig. 4). In human Ishikawa immortalised endometrial epithelial cells treated with vehicle control substantially increased expression of miRNAs (Figs. 4 and S8) however, the addition of 1000 ng/μL hPDI increased the expression of miR-324-5p, and miR-532-5p (a paralog of mir-188 originating on the Eutherian stem lineage) compared to vehicle and control (Fig. 4).

In contrast, the addition of the species-specific pregnancy recognition signals (IFNT in bovine: hCG in human) to receptive endometrial epithelial cells did not alter the expression of the core miRNA toolkit (Figs. S5 and S6 for IFNT and hCG data, respectively). These data demonstrate that the expression of the core miRNA toolkit is not altered by the species-specific pregnancy recognition signals (IFNT and hCG).

## Conclusions

Human and bovine represent two distinct implantation strategies for mammals and these lineages last shared a common ancestor some ~92 million years ago, representing ~184 million years of independent evolution^22,23^. None of the 17 miRNA genes from the 13 stem lineage miRNA families are regulated by the species-specific pregnancy recognition signals (IFNT and hCG), but the expression of the core miRNA toolkit is modified by proteins that are highly conserved amongst placental mammals (CAPG and PDI). Combined, our results show that preferential targeting of the core mammal miRNA toolkit and protein functional shift were essential to the establishment of mammalian implantation. We propose that whilst this core toolkit is present across placental mammals and is activated by conserved early pregnancy molecules, the expression levels of these miRNAs differ across species. Therefore, this core toolkit may be responsible, in part, for the diversity of implantation strategies and pregnancy morphologies observed in modern Eutheria.

In summary, this work identifies a core regulatory network that, in part, mediated the evolution of embryo implantation in mammals. With the core now defined, future work can focus on the accessory elements that facilitated the subsequent diversification of mammal implantation strategies and placental morphologies.

## Methods

### Identification of miRNAs that emerged on the eutherian stem lineage

Using the standard mammal phylogeny^47,48^ and the comprehensive set of microRNAs (miRNAs) in MirGeneDB we identified 112 miRNA families that emerged on either the eutherian or therian stem lineage^9^.

The species sampled had representation across the following vertebrate clades: *Fish*, *Amphibia*, *Reptilia*, *Aves*, *Monotremata*, *Metatheria* and *Eutheria* (as a positive control) (Table S2). For each miRNA, the stem-loop sequence (i.e. ~100nt) was used as the query sequence. Homology searches were performed in BLASTn (version 2.6.0+)^49^ with e^−10^. The phylogenetic distribution of the 112 miRNA families was determined from the patterns of presence and absence on the current mammal species phylogeny^47,48^ using the parsimony-based approach implemented in TNT^50^.

### Computational prediction of the targets of mammal stem lineage miRNAs

TargetScan^21^ was used to predict human targets for the miRNAs that were unique to, and phylogenetically conserved across, the eutheria (hereafter referred to as the “13 stem lineage miRNAs”) (Supplementary Data 1). TargetScan precomputed UTR alignments using Multiz^7^ were used for the target site predictions^21^. Only those targets predicted by TargetScan that were significantly conserved were considered in this analysis, i.e. target site sequence conservation was assessed across a diverse set of eutherian species, i.e. Human, Cow, Mouse and Elephant. Targets conserved across all four species and with the strongest binding affinity were prioritised for further analysis, i.e. 8mer-A1, 7mer-m8 and 7mer-A1 complementary binding to the seed region^51^. The protein-coding target genes were analysed for functional enrichment using PANTHER v.14^52^ and for enrichment in pathways using the Reactome Pathway Database^53^.

### Sequence conservation analysis between human and cow PDI and CAPG and their interacting partners

The level of sequence similarity between human and cow PDI and CAPG have been published previously^37^. (Note: PDI refers to the protein product of gene P4HB, PDI is a conceptus-derived protein^38^.) The interacting partners of PDI and CAPG were determined from the String network for these proteins and the human and cow orthologs were assessed for levels of sequence similarity (Table S4).

### Single Gene Ortholog annotation, alignment, and filtering for selective pressure analysis

We chose a total of 10 eutheria that demonstrate the greatest range of diversity in implantation strategy and placental morphology, plus four outgroup species one from each of the following groups: *Monotremata*, *Marsupialia*, *Aves* and *Teleostei* (Table S2). Annotated gene families were taken from Ensembl 90 (accessed 28 November 2017)^25^. A total of 7614 homologous gene families were present in all 14 species (10 placental mammals and 4 outgroups), this included paralogs. From these 7614 homologous gene families, we identified 1437 gene families that are in a single copy in the eutheria—i.e. single gene orthologs (SGOs often referred to as one-to-one orthologs), had ≥7 species^54^ and contained at least one suitable (non-eutherian) outgroup. Multiple sequence alignments (MSAs) were generated using MUSCLE v3.8.1551^55^ and MAFFT v7.310^56^ default settings and the output was compared using the ‘metal_compare’ function in VESPA^57–59^. The optimal MSA for each SGO was used for selective pressure analysis using codeml from the PAML package^60^ wrapped in the VESPA^59^ pipeline. The resolved mammal phylogeny^47,48^ was appropriately pruned for each of the 1437 SGOs using VESPA so that only those species in the alignment were in the corresponding tree for a given gene family^59^. Aligned amino acids were used in combination with the corresponding nucleotide data to generate aligned nucleotide files^59^.

### Analysis of selective pressure variation

VESPA^59^ automated the process of applying site-specific and lineage-site-specific codeml models from the PAML package^60^ across the set of 1437 SGOs, where the eutherian stem lineage was treated as foreground. We identified those putative positively selected residues on the eutherian stem lineage that were subsequently fixed in all extant Eutheria. SGOs with evidence of positive selection where the residue was fixed in all extant Eutheria were analysed for functional enrichment using GO Slim Biological Process terms and PANTHERv.14^52^. STRINGv.11 databases of interactions^61^ was used to determine if these genes interacted significantly more than expected by chance.

### Assessing if the 13 stem lineage miRNAs have significant levels of targeting to the positively selected SGOs

The 13 stem lineage miRNAs were assessed for predicted targets in the SGOs that displayed evidence of positive selection on the Eutherian stem lineage. As there were 115 SGOs under positive selection—we randomly sampled 100 sets of 115 genes that contain targets for these 13 miRNAs from the human genome but that are not positively selected (“non-positively selected” set), and we predicted targets for the 13 stem lineage miRNAs across all 100 samples. We tested our null hypothesis that there is no difference in levels of target sites predicted for the positively selected set versus the non-positively selected set of genes using a two-sample *t*-test with unequal variance, determined using Levene’s test of variance of populations^62^. The UTR length distribution between these two populations of genes was calculated.

### Modification of stem lineage miRNAS in in vitro models of early pregnancy

Unless stated otherwise, all chemicals and consumables were sourced from Sigma-Aldrich, UK.

### Bovine epithelial cell culture

The effect of P4 was assessed using cells isolated from tracts in the early luteal phase. The effect of conceptus-derived proteins was assessed in cells isolated from late luteal phase tracts to better mimic events in early pregnancy in vivo. Tracts were staged^63^ and cells isolated and cultured as previously described^37^. Briefly, the ipsilateral horns were dissected by inserting sterile curved scissors into the uterine lumen and endometrium was washed once with PBS supplemented with 1% GSP (Thermo Fisher Scientific, USA). The endometrium was dissected from the myometrium in sheets and washed in HBSS (1% GSP) and chopped into 3–5 mm fragments and digested for 1 h at 37 °C in 40 mL HBSS (10% BSA, 20 mg collagenase II, 4% DNase I, and 100X Trypsin solution). Digested tissue was passed through a 40 μM cell strainer to isolate epithelial cells, and a 70 μM strainer to isolate stromal cells. Cells were maintained in T75 flasks, in RPMI (10% FBS Gold, 1% ABAM) in a 37 °C incubator at 5% CO_2_ for 6 days, passaging every 3 days, until the cells reached 70% confluency. Cultured epithelial and stromal cells (*n* = 3 biological replicates) were counted using Trypan Blue exclusion dye. Epithelial cells were diluted to 100,000 cells/mL and plated at 2 mL/well in six-well plates prior to treatment.

Cells isolated from early luteal phase endometria were treated for 24 h with one of the following: (1) Control, (2) Vehicle (EtOH), (3) 0.1 μg/mL P4, (4) 1.0 μg/mL P4, or (5) 10.0 μg/mL P4. Cells isolated from late luteal phase endometria were treated with either: (1) control, (2) vehicle control (PBS), (3) 1000 ng/mL recombinant ovine IFNT, (4) 1000 ng/mL recombinant bovine CAPG (bCAPG), or (5) 1000 ng/mL recombinant bovine PDI (bPDI). After 24 h of treatment cells were trypsinized, pelleted, and lysed using the Invitrogen *mir*Vana miRNA extraction lysis buffer. Lysed cells were transferred to a sterile labelled tube, snap-frozen in liquid nitrogen, and stored at −80 °C.

### Human endometrial epithelial cell culture

Ishikawa cells (ECACC: #99040201) were maintained in Gibco Dulbecco’s Modified Eagle Medium: Nutrient Mixture F-12 (DMEM/F-12) (Thermo Fisher Scientific, USA) with the addition of 10% (v/v) Gibco One-shot EV-depleted FBS (Thermo Fisher Scientific, USA) (charcoal-stripped using 1 g dextran-coated charcoal) and GSP (1%). Cells were incubated at 37 °C and 5% CO_2_ in a T75 flask until they reached 70% confluency, counted using Trypan Blue exclusion dye, diluted to 100,000 cells/mL and plated at 2 mL cells/well in a six-well plate. Cells received the following treatments (*n* = 3 biological replicates): (1) control, (2) vehicle control (20 μL PBS), (3) 1000 ng/mL bCAPG or (4) 1000 ng/mL bPDI for 24 h. Cells were trypsinized, pelletted, and lysed using the Invitrogen *mir*Vana miRNA extraction lysis buffer (Invitrogen), transferred to a sterile labelled tube, snap-frozen in liquid nitrogen and stored at −80 °C. For those cells treated with hCG treatment, they were pre-treated with P4 (10 µg/mL) for 24 h, media replenished and treated with one of the following for 24 h (*n* = 3): (1) control, (2) vehicle control (10 µL ethanol, 10 µL PBS), (3) P4 only (10 µg/mL), or (4) P4 (10 µg/mL) and hCG (10 µg/mL) (Ray Biotech, USA). After 24 h cells were collected as described above.

### RNA extraction, cDNA conversion and miRNA expression analysis

Total RNA from all samples (except the hCG-treated cells which were extracted using the miRNeasy Mini Kit (Qiagen, UK)) was extracted as per manufacturer’s instructions using an acid phenol–chloroform extraction method. Following the wash steps RNA was eluted from the column into a fresh collection tube using 50 μL of nuclease-free water. Eluted RNA was DNase treated using the Invitrogen DNA-free kit, adding 5 μL 10X DNase I buffer along with 1 μL DNase I and incubated at 37 °C for 30 min. The reaction was stopped using 5 μL DNase Inactivation Reagent. Samples were mixed by pipetting and incubated at room temperature for 2 min, and centrifuged at 10,000×*g* for 15 min. The aqueous phase was collected, and RNA content was immediately quantified using the NanoDrop N1000 (Thermo Fisher Scientific, USA). Reverse transcription was performed using the miRCURY LNA reverse transcription kit (Qiagen, UK), according to the manufacturer’s instructions. RNA was diluted to 5 ng/μL using sterile DNase/RNase-free water. Two μL of diluted RNA was added to 2 μL of 5x miRCURY Reaction Buffer, 1 μL of 10× miRCURY Enzyme Mix, and 5 μL DNase/RNase free water. Samples were incubated at 42 °C for 60 min and the reaction was inactivated by incubating at 95 °C for 5 min. Quantification of miRNAs was performed using miRCURY LNA miRNA Custom PCR Panels, along with two normalisation genes and two spike-ins (Qiagen, UK; configuration #YCA21533). Cycling conditions were as follows using a Roche LightCycler (UK): 95 °C for 2 min, followed by 45 cycles (95 °C for 10 s, 56 °C for 60 s). A melting curve was included (95 °C for 1 min and 60 °C for 30 s). Delta Ct values were obtained using 5S rRNA and U6 snRNA normalisation genes. Paired 2-tailed *t*-tests were performed on dCt values in GraphPad PRISM, comparing vehicle control to treatment samples, where miRNAs were determined to be differentially expressed when *p* ≤ 0.05.

### Reactome pathway analysis of the targets of differentially expressed miRNAs

Targets of all 13 stem lineage miRNAs determined to be differentially expressed in in-vitro models of early pregnancy in human and bovine samples (Fig. 1: *p* ≤ 0.05) were converted from Ensembl transcript identifiers to gene names using Ensembl BioMart^64^. Reactome pathways were constructed from these gene names using the Reactome Pathway Database^65^ for pooled targets of all miRNAs differentially expressed in a treatment group (e.g. all significantly differentially expressed miRNAs in human Ishikawa cells treated with 1000 ng/μL bPDI).

### Statistics and reproducibility statement

Signatures of positive selection were identified in SGOs using the Vespa^59^ and Vespasian^66^ frameworks for assessing selective pressure variation analysis. These are based on the codon-based models of evolution implemented in CodeML. Log likelihood values were obtained for each model and lineage tested. The best-fitting model was determined by calculating 2Δlnl and a chi-squared test. The posterior probability of any particular codon being under positive selection is calculated using the Bayes Empirical Bayes (BEB) method^60^. STRINGDB associations were computed using methods described in^61^. Reactome Pathway analyses were performed by comparing gene sets to the full Reactome Pathway annotation of the human genome to identify statistically overrepresented pathways, with statistical results corrected for false discovery with Benjamini–Hochberg method^67^. Differences in stem lineage miRNA binding sites per transcript (PSG vs nPSG) were considered statistically significant when *p* ≤ 0.05, using a two-sample *t*-test with unequal variance, determined using Levene’s test of variance of populations^62^. miRNA qPCR expression was analysed by paired parametric 2-tailed *t*-tests performed on 2^−∆∆CT^ values in GraphPad PRISM, comparing vehicle control to treatment samples, and differential expression was reported when *p* ≤ 0.05. False Discovery rate was 1%, calculated by the Two-stage step-up method of Benjamini, Krieger and Yekutieli as recommended by GraphPad PRISM. miRNA target qPCR expression was analysed by one-way ANOVA with multiple comparisons performed on 2^−∆∆CT^ values in GraphPad PRISM, the Tukey test was used to correct for multiple comparisons, mRNA differential expression was reported when *p* ≤ 0.05.

### Reporting summary

Further information on research design is available in the Nature Portfolio Reporting Summary linked to this article.

## Supplementary information

Supplementary Information

Description of Additional Supplementary Files

Supplementary Data 1

Supplementary Data 2

Reporting Summary

## Data Availability

All data generated or analysed during this study are included in this published article or in the associated public repository at 10.5281/zenodo.7686457.
