# Supplementary material for: A burst of genomic innovation at the origin of placental mammals mediated embryo implantation
Source: Commun Biol. 2023 Apr 26;6:459. doi: 10.1038/s42003-023-04809-y (PMC10133327; doi:10.1038/s42003-023-04809-y)
Supplement: Supplementary file 1 — Supplementary Information [file 42003_2023_4809_MOESM1_ESM.pdf]

A burst of genomic innovation at the origin of placental mammals mediated embryo implantation.

Alysha S. Taylor[1,2], Haidee Tinning[1], Vladimir Ovchinnikov[3], Jessica Edge[1], William Smith[1,4], Anna L. Pullinger[1], Ruth A. Sutton[1], Bede Constantinides[2,a], Dapeng Wang[6,b], Karen Forbes[1], Niamh Forde[*1], Mary J O'Connell[*2,3]

Supplementary Figures

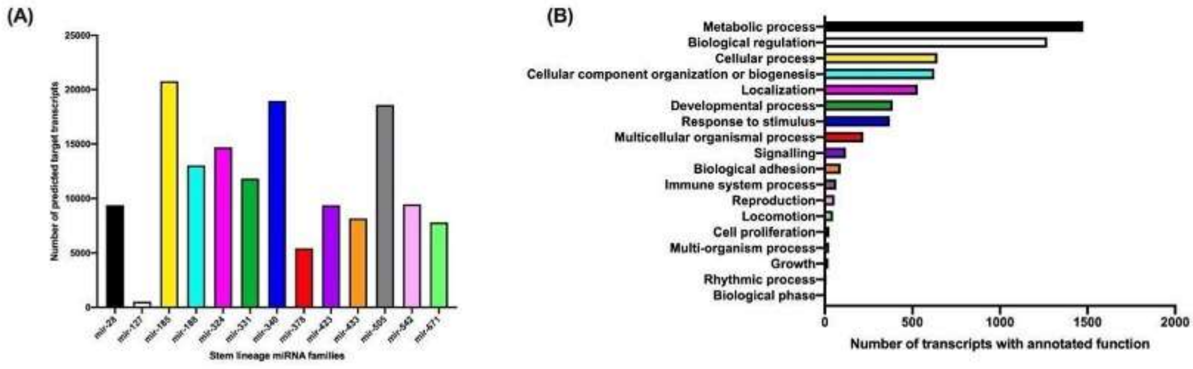

Figure S1. Number of predicted targets for each of the 13 stem lineage miRNA family and the functional annotation of the target genes using Panther DB. *(A) Targets were predicted for each of the 13 stem lineage miRNAs using TargetScan70 (Agarwal et al., 2015). TargetScan output was filtered for targets with8mer-A1, 7mer-m8 and 7mer-A1 complementary binding to the seed region. (B) Functional annotation of predicted targets of the 13 stem lineage miRNAs. Filtered stem lineage miRNA target transcripts were analysed for functional enrichment using PANTHERv.14 (Muruganujan et al., 2018), where PANTHERv.14 found functional annotations to be significantly enriched when p≤0.05.*

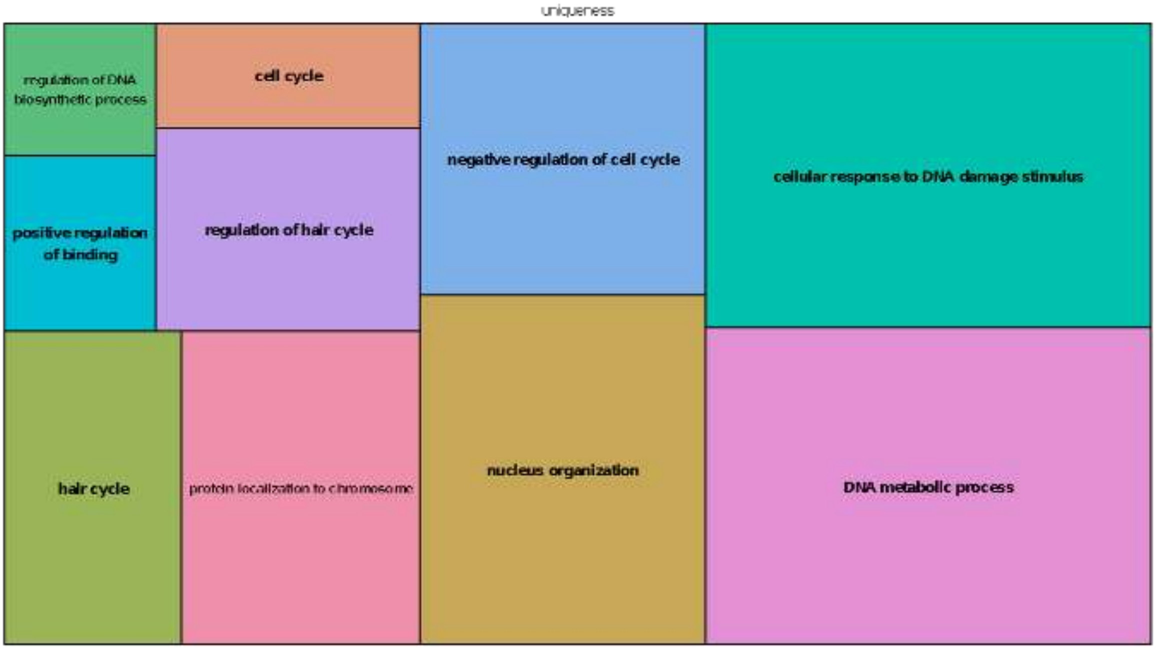

Figure S2: A standard TreeMap from REVIGO displaying the GO biological process terms present in the 84 PSGs that were predicted targets of the 13 stem lineage miRNAs. *Rectangle size represents semantic uniqueness of GO term, defined by REVIGO as the negative of average similarity to all other terms present in human.*

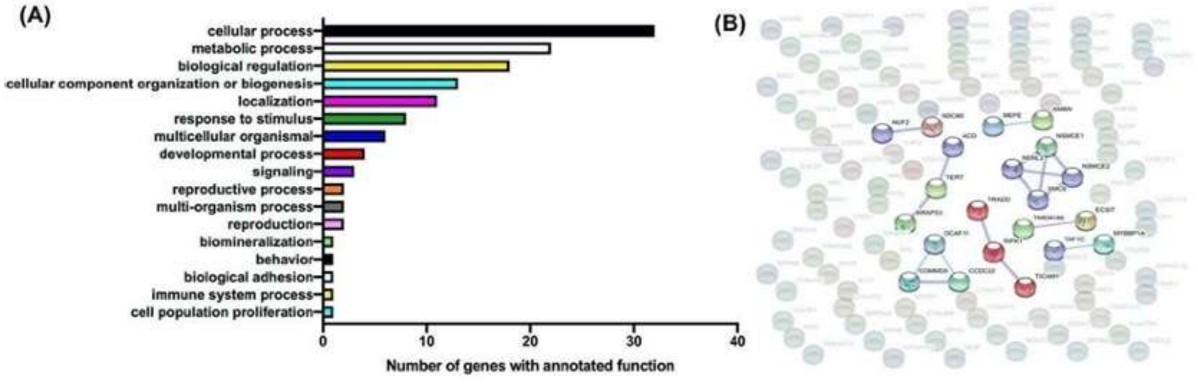

Figure S3. Broad functions of the genes with signatures of positive selection and sample of their interactions. *(A) Functional annotation using Gene Ontology Biological Process terms of 115 SGOs that underwent positive selection on the stem eutherian lineage and where the amino acid substitution was fixed on all extant Eutheria tested. The absolute number (out of 115) of positively selected genes in a given category are shown in the X axis and the functional annotations on the Y-axis. (B) String interaction network of the same set of 115 genes. Network has 106 total nodes and 8 edges (expected edges =4). Background nodes, with no high confidence interactions from experimental and database sources are faded. Nodes with high confidence interactions from experimentally determined (pink lines) or curated database (blue lines) sources are depicted in colour. The network was found to be significantly enriched for gene-gene interactions (p=0.0468). Average node degree is 0.151, with an average local clustering coefficient of 0.104. Minimum interaction score is 0.700.*

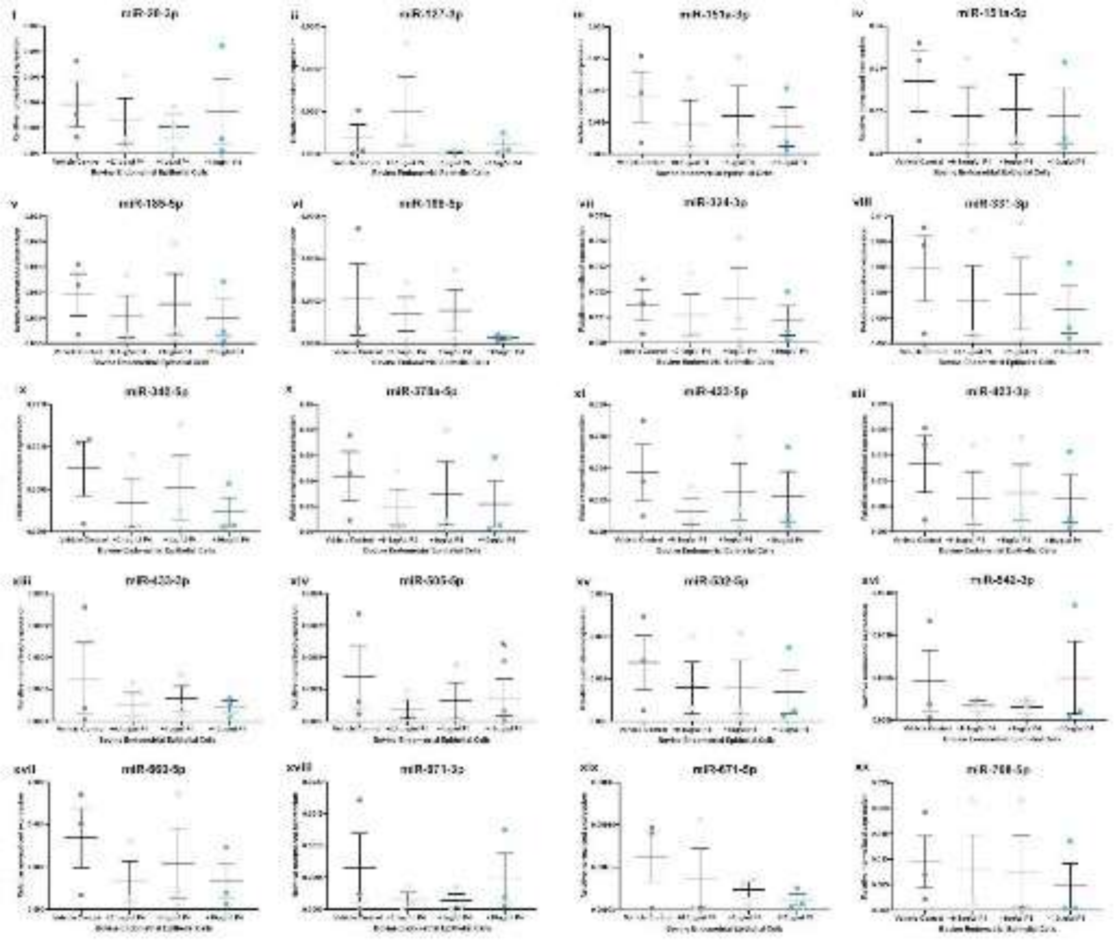

Figure S4. Expression of stem lineage miRNAs in bovine endometrial epithelial cells treated with P4. *Expression of stem lineage miRNA (i) miR-28-3p, (ii) miR-127-3p, (iii) miR-151a-3p, (iv) miR-151a-5p, (v) miR-185-5p, (vi) miR-188-5p, (vii) miR-324-5p, (viii) miR-331-3p, (ix) miR-340-5p, (x) miR-378a-5p, (xi) miR-423-3p, (xii) miR-423-5p, (xiii) miR-433-3p, (xiv) miR-505-5p, (xv) miR-532-5p, (xvi) miR-542-3p, (xvii) miR-660-5p, (xviii) miR-671-3p, (xix) miR-671-5p and (xx) miR-708-5p in bovine endometrial epithelial cells treated with vehicle control (grey circle), 0.1µg/mL (light blue), 1.0µg/mL (medium blue) or 10µg/mL P4 (dark blue circle) for 24 hours. Significant differences in miRNA expression values determined when p≤0.05 are depicted by an asterisk (*).*

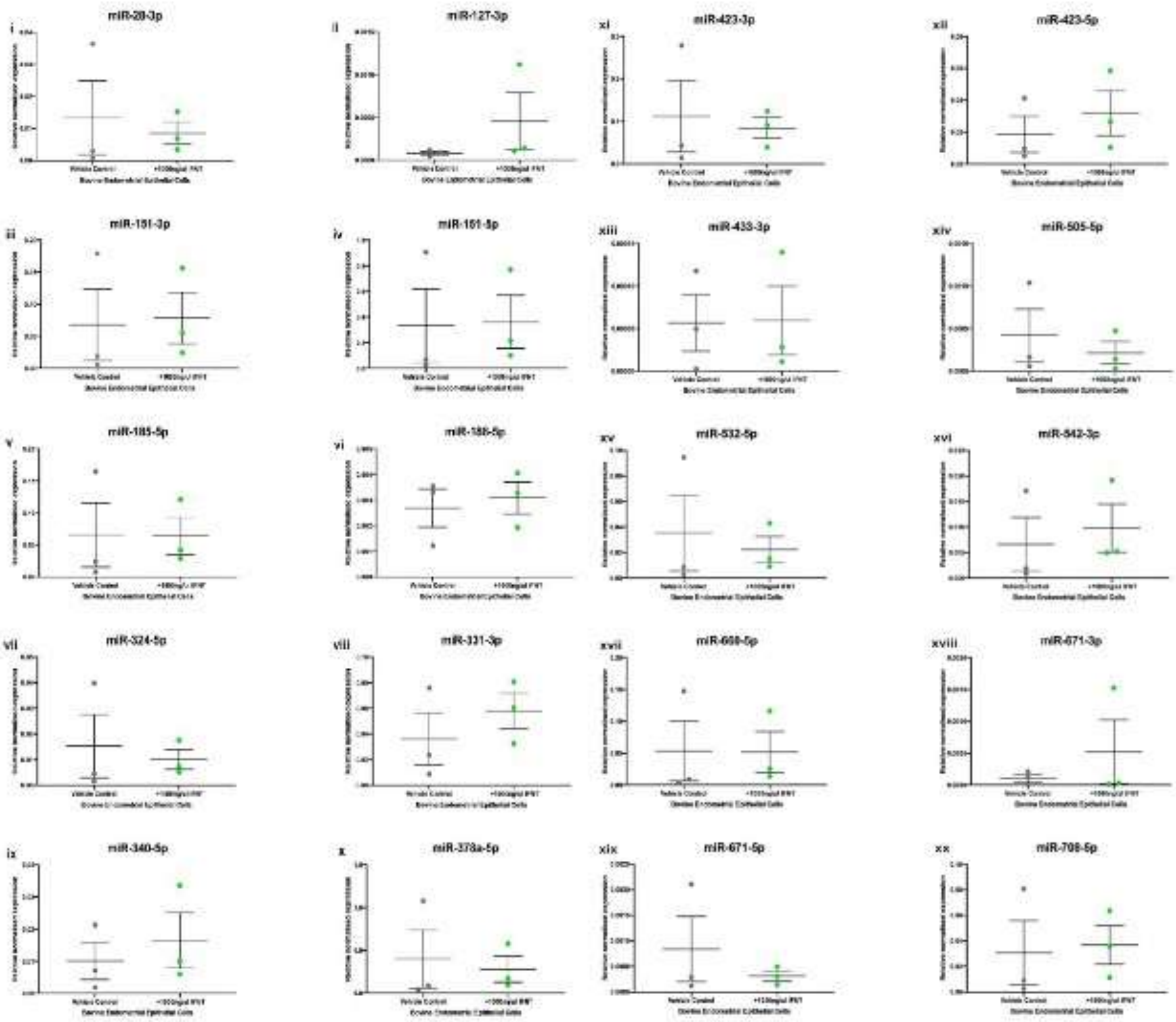

Figure S5. Expression of stem lineage miRNAs in bovine endometrial explants treated with recombinant oIFNT. *Expression of stem lineage miRNA (i) miR-28-3p, (ii) miR-127-3p, (iii) miR-151a-3p, (iv) miR-151a-5p, (v) miR-185-5p, (vi) miR-188-5p, (vii) miR-324-5p, (viii) miR-331-3p, (ix) miR- 340-5p, (x) miR-378a-5p, (xi) miR-423-3p, (xii) miR-423-5p, (xiii) miR-433-3p, (xiv) miR-505-5p, (xv) miR-532-5p, (xvi) miR-542-3p, (xvii) miR- 660-5p, (xviii) miR-671-3p, (xix) miR-671-5p and (xx) miR-708-5p in bovine endometrial explants treated with vehicle control (grey circle), or 1000ng/µl oIFNT (green circle) for 24 hours. No differences in miRNA expression were determined.*

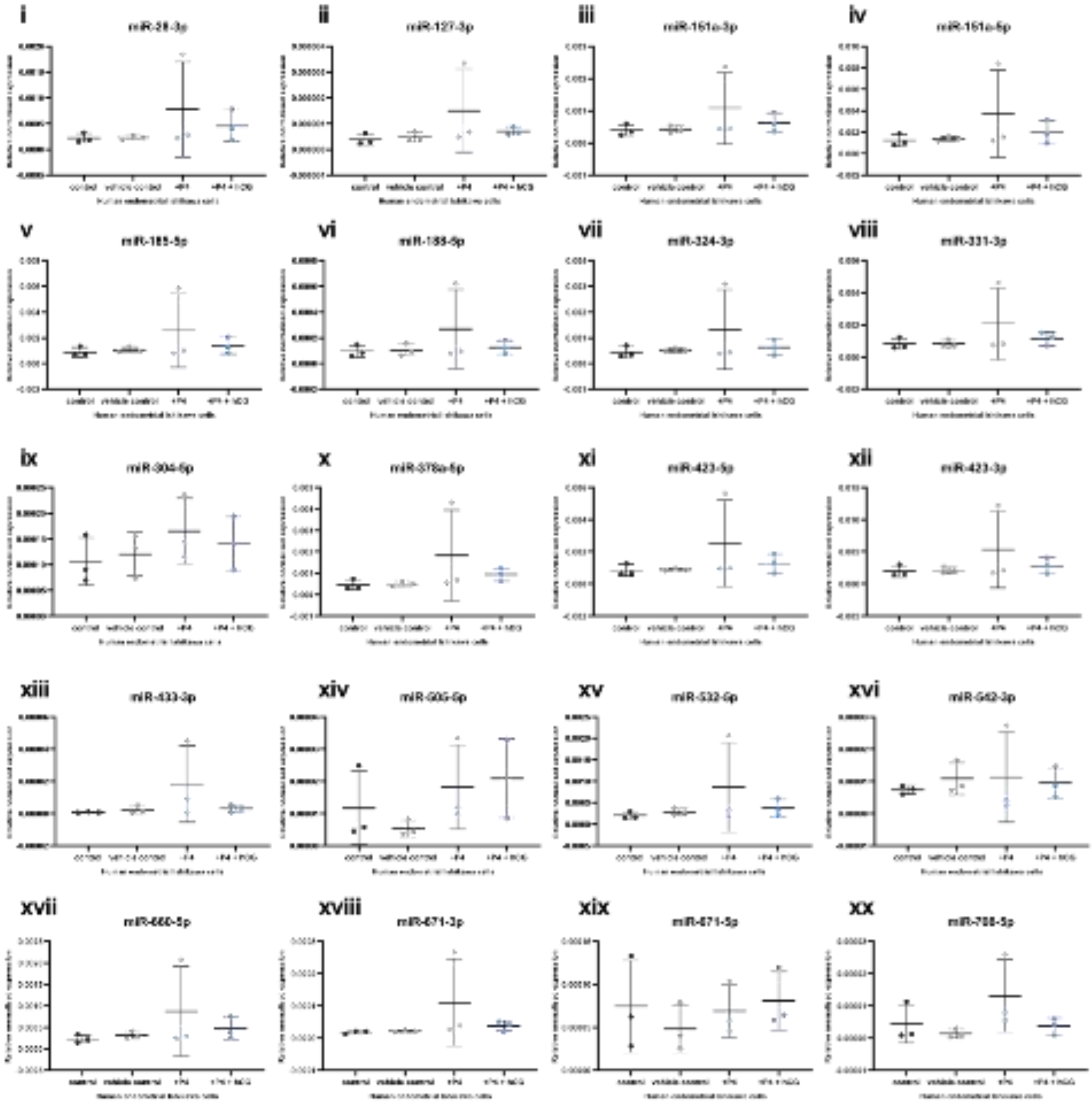

Figure S6. Expression of stem lineage miRNAs in human endometrial epithelial cells treated with hCG. *Expression of stem lineage miRNA (i) miR-28-3p, (ii) miR-127-3p, (iii) miR-151a-3p, (iv) miR-151a-5p, (v) miR-185-5p, (vi) miR-188-5p, (vii) miR-324-5p, (viii) miR-331-3p, (ix) miR-340-5p, (x) miR-378a-5p, (xi) miR-423-3p, (xii) miR-423-5p, (xiii) miR-433-3p, (xiv) miR-505-5p, (xv) miR-532-5p, (xvi) miR-542-3p, (xvii) miR- 660-5p, (xviii) miR-671-3p, (xix) miR-671-5p and (xx) miR-708-5p in human Ishikawa immortalized endometrial epithelial cells were treated with control (dark grey circle), vehicle control (light grey circle), or P4 (light blue circle), or P4+hCG (darker blue circles) for 24 hours. No differences in miRNA expression were determined.*

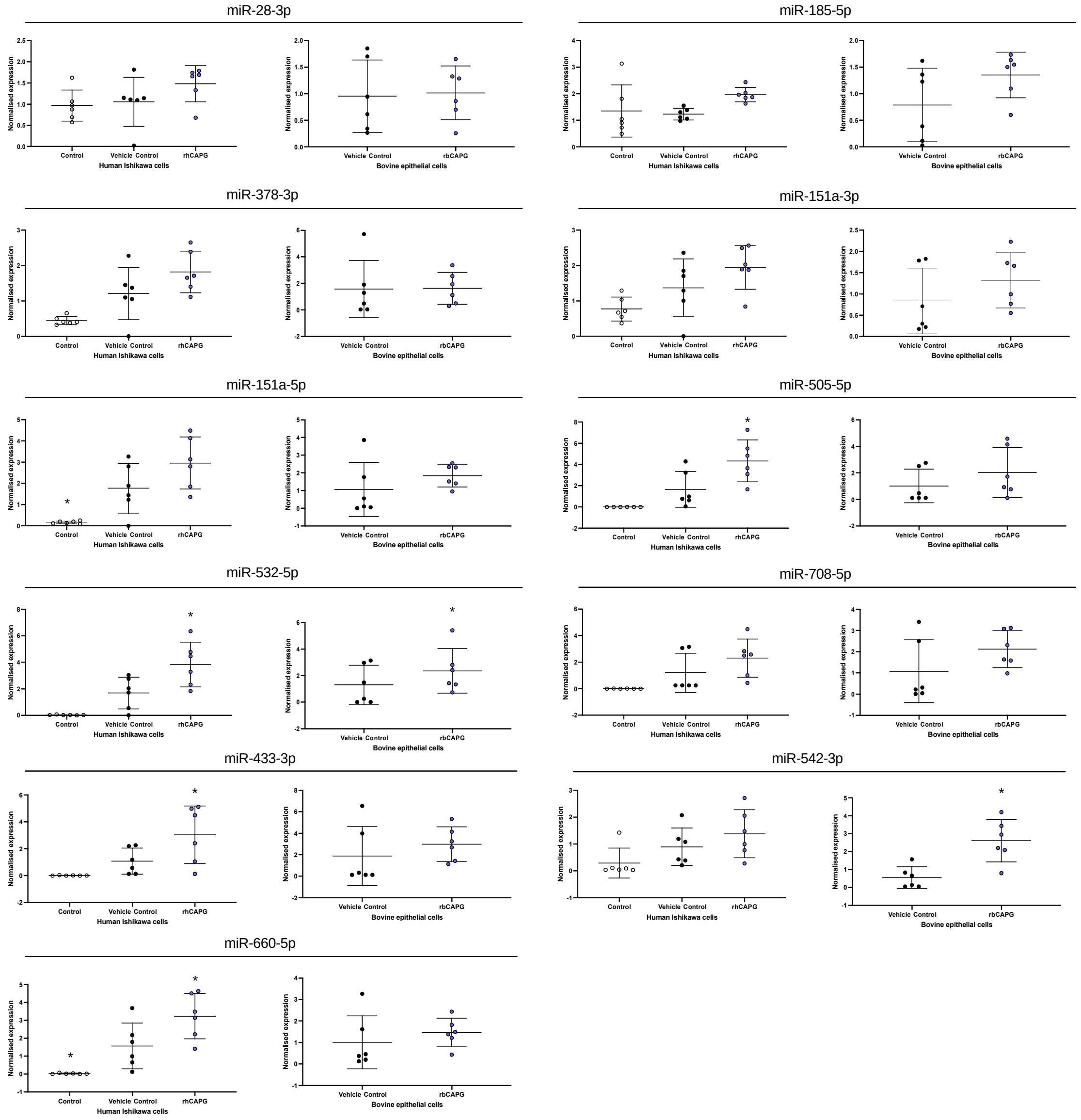

Figure S7. Expression of stem lineage miRNAs in human (left hand side) endometrial epithelial cells treated with human recombinant CAPG and bovine (right hand side) endometrial epithelial cells treated with recombinant bovine CAPG. Expression of stem lineage miRNA (i) miR-28-3p, (ii) miR-185-5p, (iii) miR-378-3p, (iv) miR-151a-3p, (v) miR-151a-5p, (vi) miR-505-5p, (vii) miR-532-5p, (viii) miR-708-5p, (ix) miR-433-3p, (x) miR-542-3p, and (xi) miR-660-5p in human or bovine endometrial epithelial cells treated with control (open circle), vehicle control (closed circle), or recombinant PDI (1000 ng/mL blue circle) for 24 hours (n=6 for all treatments). Only miRNAs with significant differences in expression ($p \leq 0.05$) are depicted with an *.

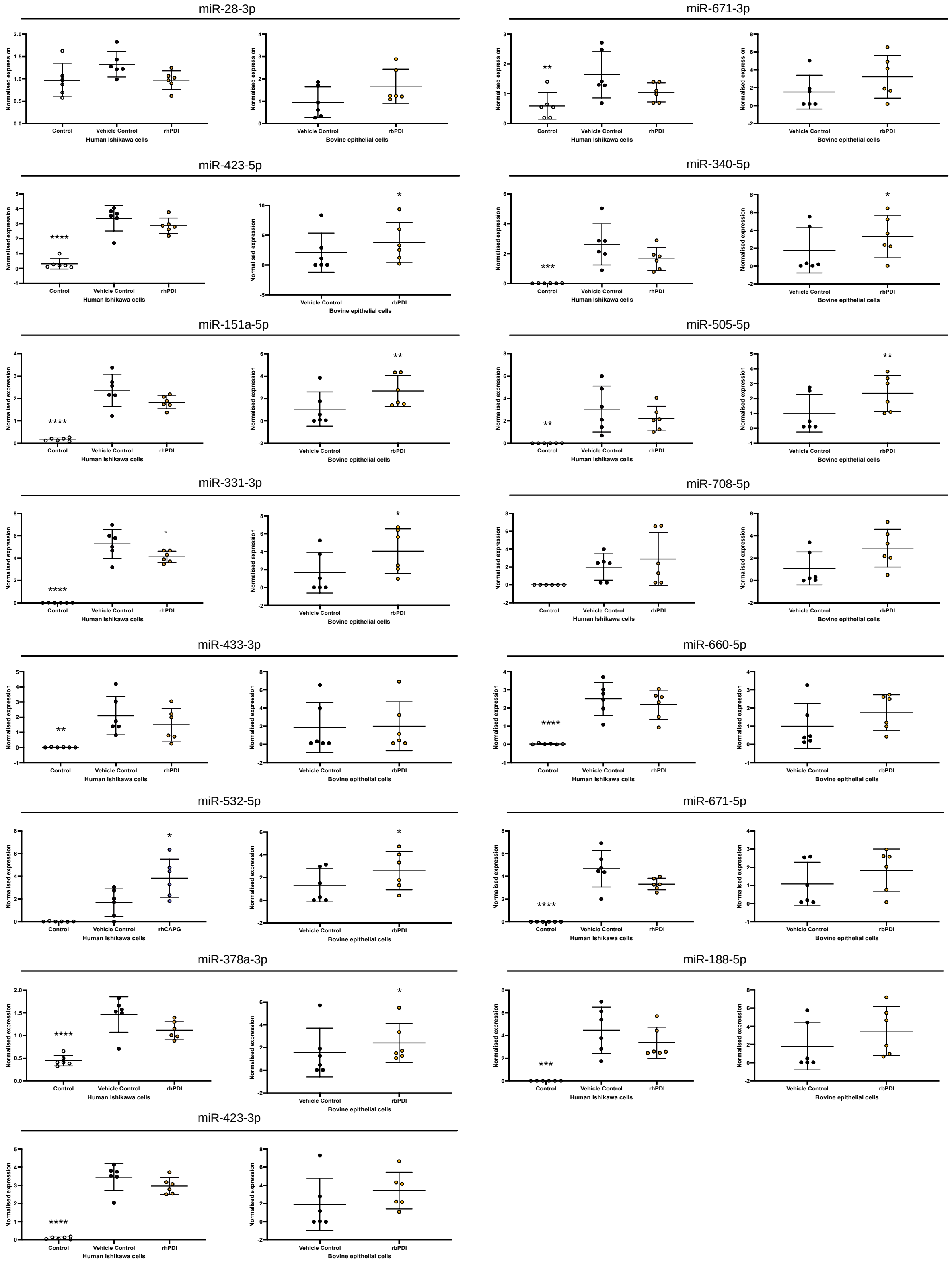

Figure S8. Expression of stem lineage miRNAs in human (left hand side) endometrial epithelial cells treated with human recombinant PDI and bovine (right hand side) endometrial epithelial cells treated with recombinant bovine PDI. Expression of stem lineage miRNA (i) miR-28-3p, (ii) miR-671-3p, (iii) miR-432-5p, (iv) miR-340-5p, (v) miR-151a-5p, (vi) miR-505-5p, (vii) miR-331-3p, (viii) miR-708-5p, (ix) miR-433-3p, (x) miR-660-5p, (xi) miR-532-5p, (xii) miR-671-5p, (xiii) miR-378a-3p, (xiv) miR-188-5p, and (xv) miR-423-3p in

human or bovine endometrial epithelial cells treated with control (open circle), vehicle control (closed circle), or recombinant PDI (1000 ng/mL blue circle) for 24 hours (n=6 for all treatments). Only miRNAs with significant differences in expression ($p \leq 0.05$) are depicted with an *.

Supplementary Tables

Supplementary Table S1: Functions for the stem lineage miRNAs outside of their direct placentation/reproduction related functions.

miRNA	Extra-placental Function
miR-28	NF-E2-related antioxidant response (Yang et al. 2011), cancer progression and migration (Lv et al., 2019; Ma et al., 2020), vascular muscle cell proliferation (Liu et al., 2021).
miR-127	Cancer progression and growth (Chang et al., 2019; Wang et al., 2018), osteoarthritis (Li et al., 2019), osteogenesis (Kuang et al., 2019), frontotemporal dementia (Piscopo et al., 2018)
miR-185	Cancer progression and migration (Wu et al., 2019), myocardial fibrosis (Lin et al., 2021), angiogenesis (Wei, J. and Zhao, Y., 2020)
miR-188	Autophagy (Wang et al., 2015), cancer cell proliferation (Luo et al., 2021)
miR-324	Renal fibrosis (Ge et al., 2019), tumor progression (Zheng et al., 2021), pulmonary hypertension (Sindi et al., 2020)
miR-331	Alzheimers disease progression (Chen et al., 2021), cancer cell proliferation (Zhao et al., 2020)
miR-340	Cancer progression and growth (Xu et al., 2021; Ren et al., 2021), intestinal inflammation (Chen, et al., 2020), vascular muscle cell proliferation (Chen et al., 2021).

miR-378	Cancer cell proliferation and migration (Zeng et al., 2017), hepatic inflammation (Zhang et al., 2018), skeletal muscle autophagy and apoptosis (Li et al., 2018).
miR-423	Cancer cell proliferation and metastasis (Ferri et al., 2022), neovascularization (Xiao et al., 2021), apoptosis and ECM degradation (Xu et al., 2020).
miR-433	Glioma progression (Zhang et al., 2021), neuronal growth and autophagy (Xu et al., 2020), coronary heart disease (Infante et al., 2019).
miR-505	Cancer cell proliferation (Li et al., 2020; Wang et al., 2021), chemokine regulation (Escate et al., 2018)
miR-542	Renal fibrosis (Li et al., 2020), neuroblastoma cell proliferation (Wei et al., 2020), chronic obstructive pulmonary disease (Farre Garros et al., 2019).
miR-671	Neuroinflammation (Deng et al., 2021), tumorigenesis (Zhu et al., 2021), rheumatoid arthritis (Chen et al., 2021)

Table S1: Stem lineage miRNAs are listed on the left, and their functions and associated citations are given on the right.

Supplementary Table S2: Vertebrate Species sampled, genome version, coverage and completion level.

Clade	Species	Version	Genome Quality
Fish	Zebrafish	GRCz11	Full Genome, Chromosome Level
	Chicken	Gallus_gallus-5.0	70X Coverage, Chromosome Level
Monotremes	Platypus	OANA5	6X Coverage, Chromosome Level
Metatheria	Opossum	monDom5	7.33X Coverage, Chromosome Level
Eutheria	Elephant	Loxafr3.0	7X Coverage, Scaffold Level
	Armadillo	Dasnov3.0	6X Coverage, Scaffold Level
	Mouse	GRCm38.p6	High Quality Reference Assembly
	Human	GRCh38.p12	High Quality Reference Assembly
	Mouse Lemur	Mmur_3.0	221.6X Coverage, Chromosome Level
	Bushbaby	OtoGar3	137X Coverage, Scaffold Level
	Cat	Felis_catus_9.0	72X Coverage, Chromosome Level
	Microbat	Myoluc2.0	7X Coverage, Scaffold Level
	Horse	Equ Cab 2	6.79X Coverage, Chromosome Level

	Cow	UMD3.1	9X Coverage, Chromosome Level

Table S2: *Set of 14 species sampled for the selective pressure analysis. Using Ensembl 92* (Yates *et al.*, 2016)*, a dataset of genomes representative of (i) vertebrate outgroup clades, or (ii) variations in placental morphology in metatherian and eutherian mammals. For each clade, genomes were chosen based on genome coverage for downstream homology searching. 'Clade' refers to the taxonomic group of each included species. 'Species' denotes the included species, by their common name. 'Version' denotes the genome assembly version included in this analysis. 'Genome Quality' refers to the coverage and assembly level of each species included in this analysis.*

Supplementary Table S3: Example of three functionally related proteins under positive selection on stem eutherian lineage

Family	Gene Name	lnL	*p*2	ω2	Positions in the alignment predicted to be positively selected.
PTHR13211	WRAP53	-10149.383	0.04821	37.80743	72, 88, 114, 225, 253, 260, 261, 331, 423, 480, 493, 506, 510, 513, 522, 528
PTHR12066	TERT	-36003.667	0.02302	954.86770	121, 122, 140, 166, 197, 508, 878, 1020, 1110
PTHR14487	ACD	-15296.106	0.10315	30.62227	138, 143, 148, 178, 185, 200, 202, 206, 208, 209, 214, 269, 278, 296, 298, 314, 347, 363, 368, 379, 390, 394, 395, 412, 414, 450, 470, 474, 524, 530, 532, 533, 545, 548, 561, 571, 575, 586, 685, 698

Table S3: *The panther family ID and common gene names are provided for a set of 3 proteins extracted to illustrate the cases of positive selection identified. The LnL value associated with the fit of the codeml model (PAML) to the data are provided as are the associated proportion*

of sites (p2) that have the corresponding $\omega 2$ (or Dn/Ds ratio). The sites estimated to be positively selected are given in the final column, these are numbered as per aligned codon position.

Supplementary Table S4: Sequence identity scores between human and bovine protein interacting partners of PDI and CAPG.

CAPG interacting partners	% identity: Human – Bovine	PDI interacting partners	% identity: Human – Bovine
CRIP1	98.70	ERO1A	95.30
UFM1	98.82	P4H4A2	94.93
PIR	92.04	SUOX	83.32
SNX3	100	MTTP	86.58
ADK	94.2	CALR	92.55

Table S4*: Interacting partners for CAPG and PDI are taken from the STRING database. The % sequence identity between human and bovine orthologs for the 10 partners are shown.*

Supplementary References:

1. Yang, M., Yao, Y., Eades, G. *et al.* MiR-28 regulates Nrf2 expression through a Keap1-independent mechanism. *Breast Cancer Res Treat* **129,** 983–991 (2011).
2. Lv, Y., Yang, H., Ma, X. *et al.* Strand-specific miR-28-3p and miR-28-5p have differential effects on nasopharyngeal cancer cells proliferation, apoptosis, migration and invasion. *Cancer Cell Int* **19,** 187 (2019).
3. Ma, L., Zhang, Y., Hu, F.miR-28-5p inhibits the migration of breast cancer by regulating WSB2. *International Journal of Molecular Medicine,* **46.4** (2020).
4. Liu Q, Yan S, Yuan Y, Ji S, Guo L. miR-28-5p improved carotid artery stenosis by regulating vascular smooth muscle cell proliferation and migration. *Vascular* (2021).
5. Suwen Chang, Liping Sun, Guijiao Feng. SP1-mediated long noncoding RNA POU3F3 accelerates the cervical cancer through miR-127-5p/FOXD1. Biomedicine & Pharmacotherapy **117** (2019).
6. Wang, D., Tang, L., Wu, H., Wang, K. and Gu, D. MiR-127-3p inhibits cell growth and invasiveness by targeting *ITGA6* in human osteosarcoma. IUBMB Life, **70** (2018)
7. Li, Z, Yuan, B, Pei, Z, et al. Circ_0136474 and MMP-13 suppressed cell proliferation by competitive binding to miR-127-5p in osteoarthritis. *J Cell Mol Med*, **23** (2019).
8. Ming-jie Kuang, Fei Xing, Dachuan Wang, Lei Sun, Jian-xiong Ma, Xin-long Ma. CircUSP45 inhibited osteogenesis in glucocorticoid-induced osteonecrosis of femoral head by sponging miR-127-5p through PTEN/AKT signal pathway: Experimental studies. *Biochemical and Biophysical Research Communications*, **509** (2019).
9. Piscopo P, Grasso M, Puopolo M, et al. Circulating miR-127-3p as a Potential Biomarker for Differential Diagnosis in Frontotemporal Dementia. Journal of Alzheimer's Disease, **65**(2):455-464 (2018).
10. Wu, W., Yu, T., Wu, Y. *et al.* The miR155HG/miR-185/ANXA2 loop contributes to glioblastoma growth and progression. *J Exp Clin Cancer Res* **38,** 133 (2019).
11. Lin, Ruizhu et al. MiR-185-5p regulates the development of myocardial fibrosis. *Journal of molecular and cellular cardiology*, **165** ,130-140 (2021).
12. Wei, Jingzan, and Yanyan Zhao. MiR-185-5p Protects Against Angiogenesis in Polycystic Ovary Syndrome by Targeting VEGFA. *Frontiers in pharmacology*, **11** (2020).
13. Luo, Zhenzhao et al. MiR-188-3p and miR-133b Suppress Cell Proliferation in Human Hepatocellular Carcinoma via Post-Transcriptional Suppression of NDRG1. *Technology in cancer research & treatment*, **20** (2021).
14. Wang, Kun et al. APF lncRNA regulates autophagy and myocardial infarction by targeting miR-188-3p. *Nature communications*, **6** (2015).
15. Ge, Yanni et al. lncRNA NR_038323 Suppresses Renal Fibrosis in Diabetic Nephropathy by Targeting the miR-324-3p/DUSP1 Axis. *Molecular therapy. Nucleic acids*, **17** (2019).
16. Zheng, Zhong et al. High miR-324-5p expression predicts unfavorable prognosis of gastric cancer and facilitates tumor progression in tumor cells. *Diagnostic pathology*, **16** (2021).
17. Sindi, Hebah A et al. Therapeutic potential of KLF2-induced exosomal microRNAs in pulmonary hypertension. *Nature communications*, **11** (2020).
18. Chen, Meng-Lu et al. Inhibition of miR-331-3p and miR-9-5p ameliorates Alzheimer's disease by enhancing autophagy. *Theranostics*, **11** (2021).

19. Zhao, Mingchuan et al. miR-331-3p Suppresses Cell Proliferation in TNBC Cells by Downregulating NRP2. *Technology in cancer research & treatment*, **19** (2020).
20. Xu, Hong-Kun et al. miR-340 Exerts Suppressive Effect on Retinoblastoma Progression by Targeting KIF14. *Current eye research*, **46** (2021).
21. Ren, Kaiming et al. MiR-340-3p-HUS1 axis suppresses proliferation and migration in lung adenocarcinoma cells. *Life sciences*, **274** (2021).
22. Chen, Qinyuan et al. miR-340 affects sauchinone inhibition of Th17 cell differentiation and promotes intestinal inflammation in inflammatory bowel disease. *Biochemical and biophysical research communications*, **526** (2020).
23. Chen, Sheng et al. MiR-340 Promotes the Proliferation of Vascular Smooth Muscle Cells by Targeting von Hippel-Lindau Tumor Suppressor Gene. *Journal of cardiovascular pharmacology*, **77** (2021)
24. Zeng M, Zhu L, Li L, Kang C. miR-378 suppresses the proliferation, migration and invasion of colon cancer cells by inhibiting SDAD1. *Cell Mol Biol Lett* (2017).
25. Zhang T, Hu J, Wang X, et al. MicroRNA-378 promotes hepatic inflammation and fibrosis via modulation of the NF-κB-TNFα pathway. *J Hepatol*. (2019).
26. Li Y, Jiang J, Liu W, et al. microRNA-378 promotes autophagy and inhibits apoptosis in skeletal muscle. *Proc Natl Acad Sci USA*. (2018)
27. Ferri C, Di Biase A, Bocchetti M, et al. MiR-423-5p prevents MALAT1-mediated proliferation and metastasis in prostate cancer. *J Exp Clin Cancer Res*. (2022)
28. Xiao Q, Zhao Y, Sun H, Xu J, Li W, Gao L. MiR-423-5p activated by E2F1 promotes neovascularization in diabetic retinopathy by targeting HIPK2. *Diabetol Metab Syndr*. (2021)
29. Xu H, Ji L, Yu C, Chen Q, Ge Q, Lu Y. MiR-423-5p Regulates Cells Apoptosis and Extracellular Matrix Degradation via Nucleotide-Binding, Leucine-Rich Repeat Containing X1 (NLRX1) in Interleukin 1 beta (IL-1β)-Induced Human Nucleus Pulposus Cells. *Med Sci Monit*. (2020).
30. Zhang J, Guo Y, Ma Y, et al. miR-433-3p Targets AJUBA to Inhibit Malignant Progression of Glioma. *Neuroimmunomodulation*. (2022)
31. Xu C, Bai Q, Wang C, et al. miR-433 Inhibits Neuronal Growth and Promotes Autophagy in Mouse Hippocampal HT-22 Cell Line. *Front Pharmacol*. (2020)
32. Infante T, Forte E, Punzo B, et al. Correlation of Circulating miR-765, miR-93-5p, and miR-433-3p to Obstructive Coronary Heart Disease Evaluated by Cardiac Computed Tomography. *Am J Cardiol*. (2019)
33. Li G, Liu F, Miao J, Hu Y. miR-505 inhibits proliferation of osteosarcoma via HMGB1. *FEBS Open Bio*. 2020
34. Wang T, Zhang H, Wang H, Chang C, Huang F, Zhang L. MiR-505-5p inhibits proliferation and promotes apoptosis of osteosarcoma cells via regulating RASSF8 expression. *J BUON*. (2021)
35. Escate R, Mata P, Cepeda JM, Padreó T, Badimon L. miR-505-3p controls chemokine receptor up-regulation in macrophages: role in familial hypercholesterolemia. *FASEB J*. (2018)
36. Li J, Bao H, Zhang K, et al. MiR-542-3p drives renal fibrosis by targeting AGO1 in vivo and in vitro. *Life Sci*. 2020
37. Wei Q, Guo Z, Chen D, Jia X. MiR-542-3p Suppresses Neuroblastoma Cell Proliferation and Invasion by Downregulation of KDM1A and ZNF346. *Open Life Sci*. (2020)

38. Farre-Garros R, Lee JY, Natanek SA, et al. Quadriceps miR-542-3p and -5p are elevated in COPD and reduce function by inhibiting ribosomal and protein synthesis. *J Appl Physiol* (2019)
39. Deng L, Guo Y, Liu J, et al. miR-671-5p Attenuates Neuroinflammation via Suppressing NF-κB Expression in an Acute Ischemic Stroke Model. *Neurochem Res*. (2021)
40. Zhu Q, Zhang X, Zai HY, et al. circSLC8A1 sponges miR-671 to regulate breast cancer tumorigenesis via PTEN/PI3k/Akt pathway. *Genomics*. (2021)
41. Chen L, Huang H, Chen L, Xu L, Chen J, Lu Q. circ-PTTG1IP/miR-671-5p/TLR4 axis regulates proliferation, migration, invasion and inflammatory response of fibroblast-like synoviocytes in rheumatoid arthritis. *Gen Physiol Biophys*. (2021).
42. Ferri, C., Di Biase, A., Bocchetti, M. *et al.* MiR-423-5p prevents MALAT1-mediated proliferation and metastasis in prostate cancer. *J Exp Clin Cancer Res* (2022).
