# Supplementary material for: A burst of genomic innovation at the origin of placental mammals mediated embryo implantation
Source: Commun Biol. 2023 Apr 26;6:459. doi: 10.1038/s42003-023-04809-y (PMC10133327; doi:10.1038/s42003-023-04809-y)
Supplement: Supplementary file 2 — Description of Additional Supplementary Files [file 42003_2023_4809_MOESM2_ESM.pdf]

Description of Additional Supplementary Files

File Name: Supplementary Data 1

Description: List of miRNAs in the “embryo implantation toolkit” with their miRbase and miRGeneDB identifiers. (Excel Spreadsheet).

File Name: Supplementary Data 2

Description: Transcripts that gained uterine expression on the therian or eutherian lineage that are predicted targets of the stem lineage miRNAs. (Excel Spreadsheet).
