# Supplementary material for: A burst of genomic innovation at the origin of placental mammals mediated embryo implantation
Source: Commun Biol. 2023 Apr 26;6:459. doi: 10.1038/s42003-023-04809-y (PMC10133327; doi:10.1038/s42003-023-04809-y)
Supplement: Supplementary file 5 — Reporting Summary [file 42003_2023_4809_MOESM5_ESM.pdf]

nature portfolio

Corresponding author(s): Forde and O'Connell

Last updated by author(s): April 1st 2023

Reporting Summary

Nature Portfolio wishes to improve the reproducibility of the work that we publish. This form provides structure for consistency and transparency in reporting. For further information on Nature Portfolio policies, see our Editorial Policies and the Editorial Policy Checklist.

Statistics

For all statistical analyses, confirm that the following items are present in the figure legend, table legend, main text, or Methods section.

n/a	Confirmed	
☐	☒	The exact sample size (*n*) for each experimental group/condition, given as a discrete number and unit of measurement
☐	☒	A statement on whether measurements were taken from distinct samples or whether the same sample was measured repeatedly
☐	☒	The statistical test(s) used AND whether they are one- or two-sided<br>*Only common tests should be described solely by name; describe more complex techniques in the Methods section.*
☒	☐	A description of all covariates tested
☐	☒	A description of any assumptions or corrections, such as tests of normality and adjustment for multiple comparisons
☐	☒	A full description of the statistical parameters including central tendency (e.g. means) or other basic estimates (e.g. regression coefficient) AND variation (e.g. standard deviation) or associated estimates of uncertainty (e.g. confidence intervals)
☐	☒	For null hypothesis testing, the test statistic (e.g. *F*, *t*, *r*) with confidence intervals, effect sizes, degrees of freedom and *P* value noted<br>*Give P values as exact values whenever suitable.*
☒	☐	For Bayesian analysis, information on the choice of priors and Markov chain Monte Carlo settings
☒	☐	For hierarchical and complex designs, identification of the appropriate level for tests and full reporting of outcomes
☒	☐	Estimates of effect sizes (e.g. Cohen's *d*, Pearson's *r*), indicating how they were calculated

Our web collection on statistics for biologists contains articles on many of the points above.

Software and code

Policy information about availability of computer code

Data collection	Data was extracted from published and publicly available databases (miRGeneDB and Ensembl)
Data analysis	Data were analysed using published and freely available software, i..e. BLAST, TargetScan, PANTHER, MUSCLE, MAFFT, VESPA, and StringDB.

For manuscripts utilizing custom algorithms or software that are central to the research but not yet described in published literature, software must be made available to editors and reviewers. We strongly encourage code deposition in a community repository (e.g. GitHub). See the Nature Portfolio guidelines for submitting code & software for further information.

Data

Policy information about availability of data

All manuscripts must include a data availability statement. This statement should provide the following information, where applicable:

- Accession codes, unique identifiers, or web links for publicly available datasets
- A description of any restrictions on data availability
- For clinical datasets or third party data, please ensure that the statement adheres to our policy

We have placed all data generated or analysed in this study in a zenodo repository online for free access to all, with the following DOI 69. DOI:10.5281/zenodo.7686457. Everything we have used is either freely available (accessions and identifiers supplied) or is described in full in the manuscript.

Human research participants

Policy information about studies involving human research participants and Sex and Gender in Research.

Reporting on sex and gender	not applicable
Population characteristics	not applicable
Recruitment	not applicable
Ethics oversight	not applicable.

Note that full information on the approval of the study protocol must also be provided in the manuscript.

Field-specific reporting

Please select the one below that is the best fit for your research. If you are not sure, read the appropriate sections before making your selection.

☒ Life sciences ☐ Behavioural & social sciences ☐ Ecological, evolutionary & environmental sciences

For a reference copy of the document with all sections, see nature.com/documents/nr-reporting-summary-flat.pdf

Life sciences study design

All studies must disclose on these points even when the disclosure is negative.

Sample size	To generate the biological replicates, we chose n=3 as per Tinning et al 2020.
Data exclusions	none of the samples were excluded (see Fig 3&4).
Replication	we included n=3 replicates.
Randomization	not applicable
Blinding	not applicable

Reporting for specific materials, systems and methods

We require information from authors about some types of materials, experimental systems and methods used in many studies. Here, indicate whether each material, system or method listed is relevant to your study. If you are not sure if a list item applies to your research, read the appropriate section before selecting a response.

Materials & experimental systems

n/a	Involved in the study	
☒	☐	Antibodies
☐	☒	Eukaryotic cell lines
☒	☐	Palaeontology and archaeology
☒	☐	Animals and other organisms
☒	☐	Clinical data
☒	☐	Dual use research of concern

Methods

n/a	Involved in the study	
☒	☐	ChIP-seq
☒	☐	Flow cytometry
☒	☐	MRI-based neuroimaging

Eukaryotic cell lines

Policy information about cell lines and Sex and Gender in Research

Cell line source(s)	Human ishikawa cell lines as follows: ECACC 99040201. primary bovine cells were derived from waste tissue from abbattoir.
Authentication	Ishikawa cells (ECACC: 99040201) were maintained in Gibco Dulbecco's Modified Eagle medium: Nutrient Mixture F-12 (DMEM/F-12) (Thermo Fisher Scientific, USA) with the addition of 10% (v/v) Gibco Pne-shot EV_depleted fBS (ThermoFisher Scientific, USA)(charcoal stripped using 1g dextran-coated charcoal) and GSP (1%). Bovine cells were previously authenticated as in Tinning et al (2020) bt FACS analysis.

Mycoplasma contamination	all tested negative.
Commonly misidentified lines<br>(See ICLAC register)	not applicable.
